# Radiation characteristics of natural gamma-ray from coal and gangue for recognition in top coal caving

**DOI:** 10.1038/s41598-017-18625-y

**Published:** 2018-01-09

**Authors:** Ningbo Zhang, Changyou Liu

**Affiliations:** 1 School of Information and Electrical Engineering, China University of Mining and Technology, Xuzhou, 221116 China; 2 School of Civil, Mining and Environmental Engineering, University of Wollongong, Wollongong, New South Wales 2522 Australia; 3 State Key Laboratory of Coal Resources and Safe Mining, China University of Mining and Technology, Xuzhou, 221116 China; 4 School of Mines, Key Laboratory of Deep Coal Resource Mining, Ministry of Education, China University of Mining and Technology, Xuzhou, 221116 China

## Abstract

Recognition of coal and gangue (roof rock) is a key technology to realize fully mechanized top coal caving automated mining. This paper proposes to detect the instantaneous refuse content of drawn coal and gangue mixture during top coal caving by using natural gamma-ray technology. The generating environment of coal and rock seams, the distribution characteristics of natural gamma ray from coal and roof-rock and the principle of coal-gangue recognition using natural gamma-ray method were analyzed. The natural gamma ray radiation characteristics of coal and roof-rock seams from seven different typical coal mine areas who has thick coal seams in China have been researched, and a connection between radiation intensity and refuse content was set up. The experiments on the mixed condition of roof-rock drawn from caving opening in the caving process of fully-mechanized top coal caving working face was taken and the radiative signals was real-time detected by using the self-developed coal-gangue recognition experimental system. The experiments results demonstrate the feasibility of using natural gamma-ray technology to perform real-time detection of refuse content of drawn coal and gangue mixture and the availability of self-developed coal-gangue recognition detector.

## Introduction

In China, coal is the main energy source, accounting for about 70% of primary energy production and consumption^1,2^. Since top coal caving mining method has the advantage of high-production, high-efficiency, low development ratio, and low-coast, it has developed rapidly in the past two decades and become the most extensively used mining method in China^3–7^. However, the recovery ratio and quality of top coal are optical because that the top coal caving process is completely dependent on the experience of caving workers to discriminate coal or gangue’s whereabouts and operating hydraulic support to complete control, inevitably, “over” and “less” situation was caused. Consequently, the coal-gangue recognition, i. e, detect the instantaneous refuse content of drawn coal and gangue mixture during top coal caving process, should be researched to realize the automated top coal caving who is the key to automated fully mechanized top coal caving mining of thick and extra-thick coal seams^8–13^.

The methods used for coal-gangue recognition such as tail beam vibration detection,γ-ray detection, radar detection, cutting force response, infrared detection, and image detection have been investigated by researchers in recent years^14^. In detail, the marginal spectrum and frequency spectrum of the acoustic signals collected in fully-mechanized caving face were analyzed to identify the coal-rock interface^15^; the time-domain statistical feature parameters method was used to analyze the vibration signal caused by coal, gangue and roof rock caving and impacting the hydraulic support tail beam and the rear scraper conveyor to solve the coal and rock character recognition method in the top coal caving process^16^; the MATLAB software was used to analyze the coal rock image signals for image processing technology of coal and rock characters identification^17^; the coal gangue acoustic signals were analyzed based on sparse representation^18^; and the tail beam vibration signals, investigated by an increasing number of researchers in recent years and used in many practical applications, were measured by using acceleration sensors to identify the rock–coal interface^8,9,19,20^.

These methods above have acquired some valuable achievements and have been applied in several fields. However, such techniques present with disadvantages. Artificial γ rays are harmful to human beings. Meanwhile, radar detection faces the problem of considerable contradiction between measuring range and precision. Meanwhile, cutting force response method is unsuitable for the top coal caving mining environment, whereas infrared detection method is insufficiently mature. In addition, the recognition accuracy rate of the above-mentioned methods is highly sensitive to the coal mine environment^14^.

In this study, we apply natural gamma-ray method to realize the automatic recognition of coal and gangue. Using this method means to determine the intensity of γ-ray from the natural radioactive matters contained in the material drawn out from the caving opening and then, based on the intensity, determines the material’s refuse content. In sedimentary strata, radioactive matters naturally exist^21–24^. The content of radioactive material in gangue is much greater than that of coal, and the content of radioactive material in coal is so lower that it can be ignored^25–29^. That means the content of radioactive material in gangue and coal is quite different, so the refuse content in drawn coal and gangue mixture can be determined by measuring the mixture’s radiation. This provides a theoretical basis for the automatic top coal caving technology in fully mechanized caving mining. At the same time, the natural ray method for coal and gangue recognition has fast speed, non-contact measurement, not affect other equipment and so on.

## Results

### Radiation characteristics of coal and roof-rock samples

We can know from the analysis that the content of the radionuclide in the sedimentary rocks is related to the content of clay minerals, the formation time, the environment of the sedimentary area and so on^30–32^, therefore, we carried out analysis on the radiation characteristics of the coal and roof rock in the thick and extra-thick coal seam for the selected typical mining areas such as Dongsheng, Datong, Yanzhou, Shuozhou and Longkou^33–36^. And the selected Coal Mine are Suancigou (SCG), Tongxin (TX), Xinzhouyao (XZY), Pingshuo NO.2 (PS.2), Xinglongzhuang (XLZ), Nantun (NT) and Beizao (BZ).

**Figure 1:**
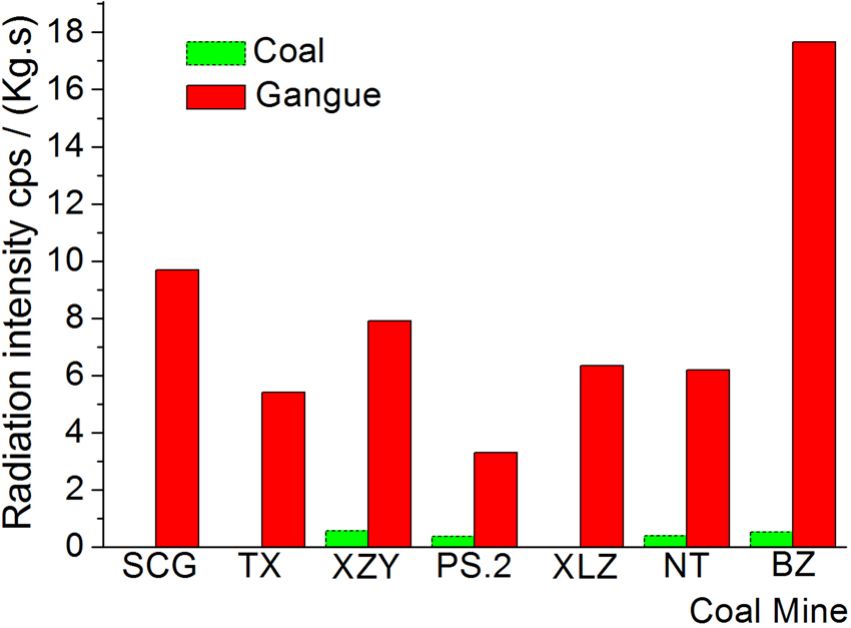
Radiation characteristics of coal and roof rock in different coal mines.

The radiation characteristics of the coal and gangue samples of the investigated mines were measured and recorded in Fig. 1. In the measuring process, the shielding effect of coal samples of SCG, TX and XLZ on environment background radiation is greater than their own contribution, so the radiation value is set to be 0.

As can be seen from Fig. 1, the radiation intensities of all coal samples are generally small, even less than their own shielding ability, so it is considered that the radiation content of coal is 0 in the research process of this paper. The roof rock radiation intensity is much bigger than that of coal samples, but the difference is great between different gangues, the average radiation intensity is 8.08 cps/(Kg.s). The gangue radiation intensity in BZ Coal Mine (belongs to the offshore-type of the Tertiary period and has short diagenetic time) is the largest, reaching to 17.67 cps/(Kg.s), the roof belongs to the shale with strong adsorption capacity, therefore, it has large content of radioactive material. The minimum gangue radiation intensity of PS.2 Coal Mine is 3.31 cps/(Kg.s). For the rock with different lithology in the same period, the radiation intensity is directly related to grain size. The smaller the particle size, the greater the radiation intensity.

The sample radiation intensities from the same layer of sedimentary rock of the same coal field have little difference. In Fig. 1, the rock sample from XLZ and NT Coal Mine of Yanzhou coal field are siltstone, their radiation value difference is very small, which is only 0.15 cps/(Kg.s). The radiation intensities of different types of rock strata in the same coal field have large difference. The radiation intensity difference value of the fine sandstone and sandy shale form TX Coal Mine and XZY Coal Mine of Datong Coal field reached 2.48 cps/(Kg.s). This has provided basis for the radiation intensity calibration of automatic recognition technology in fully mechanized caving mining.

### Experimental result analysis

#### Background radiation calibration of experimental environment

**Figure 2:**
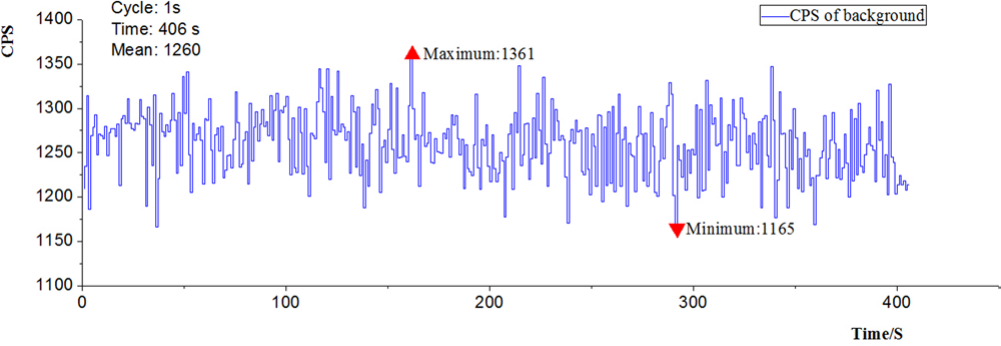
CPS curves of environment background radiation.

In order to eliminate the influence of experimental environment on the measurement and get the real radiation value of coal and gangue, the background radiation of the experimental environment is tested, the Fig. 2 is the measured data distribution curve.

#### Measurement of radiation quantity of crushed gangue

**Figure 3:**
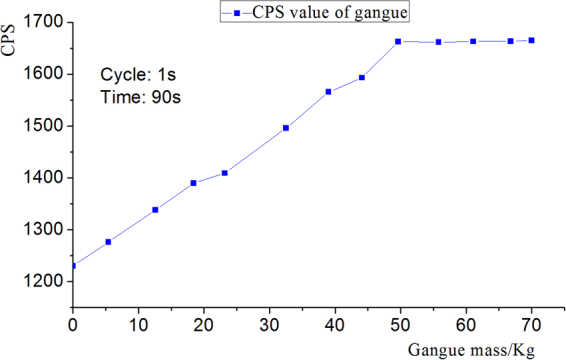
Relationship between mass of gangue sample and average CPS value.

In order to measure the radiation intensity of gangue and its relationship with mass, gangue of different mass were placed under the probe, and its radiation quantity was measured. Probe sampling period was 1000 ms, the measurement time of each sample was 90 s. Each gangue cps value was the mean value of 90 measurement data in measuring time. Figure 3 is the relationship between mass of gangue samples and average CPS value.

As shown in Fig. 3, the gangue radiation values were determined. When the gangue content is 18.4 Kg, the radiation quantity has reached 13% of the environment background radiation, indicating that the detector has high resolution ratio for gangue. The increase of CPS value is basically linear with the increase of rock samples quantity. When the gangue mass reaches a certain value, the increase of radiation CPS value is not obvious, i.e., it is close to the radiation saturation thickness. When the measured value is approximately equal to the threshold value of radio activity recognition, the emergence of gangue can be obviously judged from the figure. Therefore the measured gangue content in the coal and gangue mixture at the coal caving opening by natural ray technique is credible.

#### Result of top coal caving simulation test

**Figure 4:**
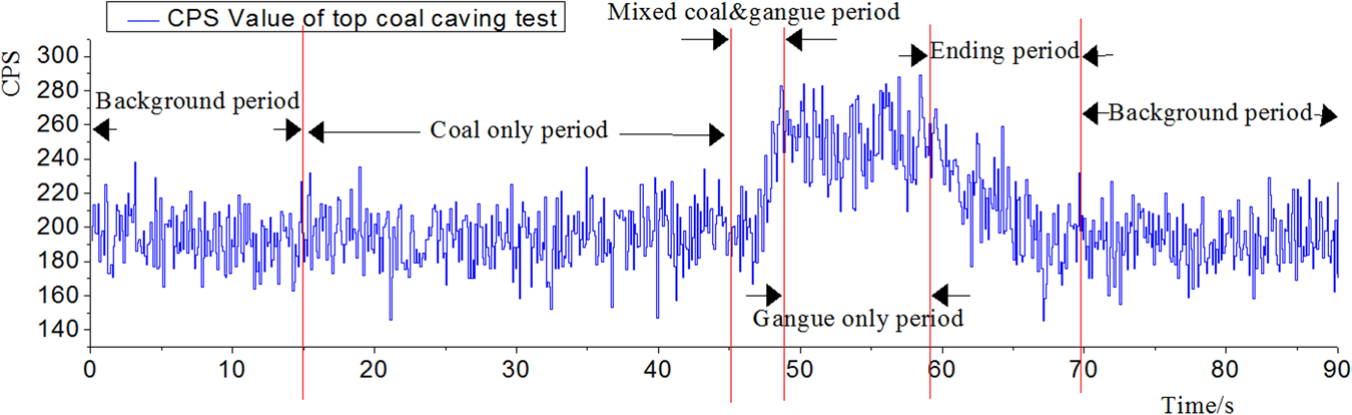
The measured radiation intensity of gangue.

Figure 4 is the variation curve of coal and gangue radiation intensity detected by the detector in the caving process of coal and rock simulated by the coal-gangue recognition test platform.

It can be seen from the Fig. 4 that after opening the coal caving opening (start of coal only period), the radiation intensity is rising rapidly after a period of stable status, indicating that the crushed coal which stream out when the coal caving opening just opened almost has no radioactivity. When the discharges of coal caving opening gradually change from the coal only to coal and gangue mixture and finally to gangue only, the radiation intensity detected by the detector is increased with the increase of the gangue content, this process lasts for 4.4 s. The radiation intensities remain stable until the rock is not enough, then the radiation intensity decreases with the gradual reduction of the roof rock in the funnel, and the detected radiation curve steadily fell to the background value.

## Discussion

Coal-gangue recognition is a bottleneck technology to realize the automation of fully mechanized top coal caving. At present, a large number of scholars have studied it in different ways, but the real-time performance and the veracity of recognition are still to be further ascension. It is found that coal and gangue are generally radioactive, the detector for natural radiation was developed, and through laboratory tests, the radiation intensity of coal and gangue is very different. The radionuclide content is similar for the same kind of rocks; radionuclide content has great difference between different kinds of rocks; in the coal bearing series, the radioactive intensity decreases with the increasing of the particle size and decreases with the increase of the diagenetic time. And, most importantly, the natural radiation value of coal sample is almost zero, which can be ignored in the measurement process; the quantity of gangue radiation increases linearly with the mass; when the gangue content reaches a certain value, the radiation quantity has no obvious increase. The increase of gangue quantity after reaching the saturation of radiation has no contribution for the radiation detection by the probe. The radiation value can be accurately measured and judged in a short period of time before the gangue content reaches saturation, which makes it possible to determine and identify the gangue content in coal and gangue mixture. Therefore, radiation characteristics difference of natural gamma-ray from coal and gangue was used to realize recognizing the gangue from the drawn mixture of coal and gangue.

Through theoretical analysis we can know: The radiation intensity of coal and gangue mixture discharged from the coal caving opening has a one-to-one corresponding relationship with the mixed degree of gangue, which means that the interfusion situation of gangue can be obtained through monitoring the change of radiation intensity at the coal caving opening.

At the same time, the coal-gangue recognition test platform was developed to simulating the process of top coal caving and detecting the radiation signal from the coal and gangue drawn out of the top coal caving opening. The experiment result shows that: using natural ray technique to identify coal and gangue in the top coal caving process can achieve the goal of accuracy and real-time response as well as the correct guidance of coal caving window operation, providing a reliable basis for recognition of the automated top coal caving technology.

The results of top coal caving simulation test verify the reliability of coal and gangue recognition by means of natural ray technology, this technology can accurately detect the changes of gangue content in coal and gangue stream of the coal caving opening and have real-time response, making guidance for top coal caving operation.

Experiments in this paper is just simulating a single structure coal seam of fully mechanized top coal caving mining process for testing the recognition of coal and gangue, the effect from the parting in complex structure thick coal seam, the floor rock and the gangue from gob was not took into account. At the same time, the reasonable position for detector, the temperature and humidity of the caving space and the matching between the recognition index and support controlling still needed to be further researched and practiced.

## Methods

### Formation and distribution of radionuclides in coal and rock strata

Coal bearing series (coal measures for short) is a series of sedimentary rock or strata containing coal or coal seam. All kinds of rocks contain a certain amount of radionuclides in nature, so does the coal bearing series [12].

For the coal mine roof, the different sedimentary rocks have different radiation characteristics. The content of radionuclides in the roof is mainly related to the grain size of the sediments, the quantity of organic matter, sedimentary environment and conditions, and the deposition time. There are the following general rules^[12]^:

(1) The same type of rocks has similar content of radionuclide, while radionuclide content has great difference between different kinds of rocks; (2) In coal bearing series, the radioactive intensity is the lowest in coal, while it gradually increases in the conglomerate, grit stone, medium sand, fine sandstone, siltstone, silty mudstone, shale and mudstone,. The smaller the particle size, the greater the mud content and the stronger the radiation; (3) The radiation of inland-type gangue is less than that of offshore-type gangue. (4) The shorter the coal forming time, the stronger the roof radiation. The thick coal seams are mostly lignite with a low degree of metamorphism, the roof formation time is less than bituminous coal and anthracite coal, so the radiation of the roof is relatively large. Therefore for similar gangues, the radiation intensity increases with the shortening of time. This is advantageous to the application of the natural ray method to identify coal and gangue. (5) The radiation content is large in the sedimentary rocks containing diagenetic minerals such as leopoldite. In the determination of the radiation of coal mine roof, it is need to first determine the composition of its diagenetic mineral, so as to facilitate the recognition of the source of natural radiation.

### Determination of Research coal mines

**Figure 5:**
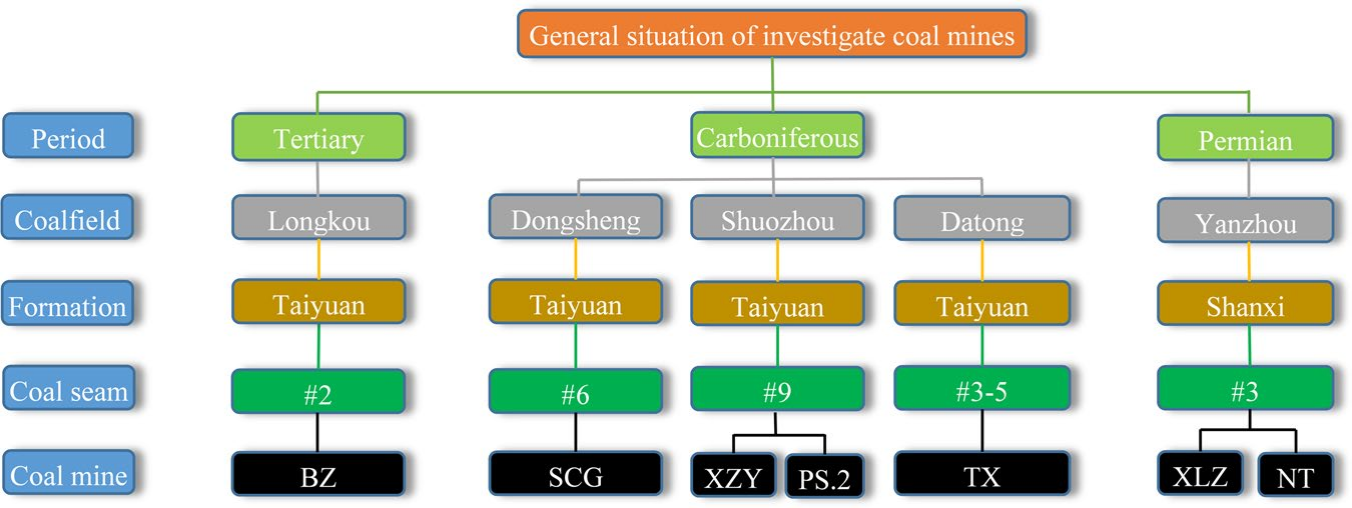
The investigated coal mines and their general situation.

We can know from the above analysis that the content of the radionuclide in the sedimentary rocks is related to the content of clay minerals, the formation time, the environment of the sedimentary area and so on, therefore, we carried out analysis on the radiation characteristics of the coal and gangue in the thick and extra-thick coal seam for the selected typical mining areas such as Dongsheng, Datong, Yanzhou, Shuozhou and Longkou. The research details are shown in Fig. 5. And the roof lithologic is shown in the Table 1.

**Table 1:** Lithologic statistics of roof.

Coal Mine	SCG	TX	XZY	PS.2	XLZ	NT	BZ
Coal type	Durain	Semibright	Bright	Half bright and half dark	Semibright	Durain	Lignite
Roof lithology	Sandy mudstone	Fine sandstone	Sandy shale	Grit stone	Siltstone	Siltstone	Oil-bearing mudstone

### The principle and technique of automatic recognition of coal and gangue

The coal and gangue recognition technology by means of natural ray determines the gangue content in coal and gangue mixture stream based on the natural radiation intensity of gangue. The measured data is not influenced by the coal/gangue size, or the environment around the coal caving opening, therefore it has certain advantages. Overall, at the coal caving opening of a top coal caving face, the refuse rate of the drawn-out mixture is zero at first, and then increases gradually and continuously. Therefore, the trend of gangue discharge can be determined based on the instantaneous radiation intensity of the mixture stream.

### Determination of gangue content in coal and gangue mixture

**Figure 6:**
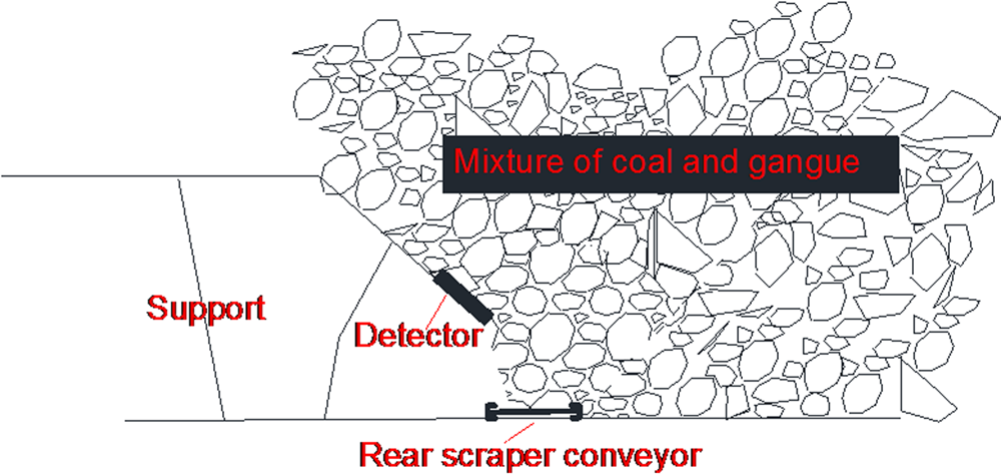
Work mechanism of the detector.

**Figure 7:**
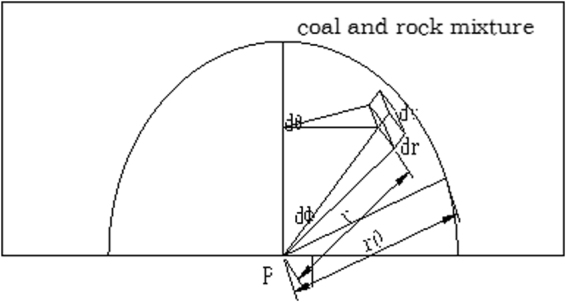
Coal and gangue mixture calculation model.

As seen in Fig. 6, the detector’s probe is placed above the support shield beam. Based on this configuration, the coal and gangue detection calculation model is established as shown in Fig. 7, where point P marks the detector.

For easiness of discussion, we assume that the coal and gangue mixture is a uniform mixture, and then the mass attenuation coefficient of the mixture is

1 $$\mu ={\mu }_{{\rm{c}}}{c}_{c}+{\mu }_{{\rm{g}}}{c}_{g}={\mu }_{{\rm{c}}}+({\mu }_{{\rm{g}}}-{\mu }_{c}){c}_{g}$$

where *μ*_c_ is the mass attenuation coefficient of coal, *c*_*c*_ is the mass percentage content of coal in the mixture, *μ*_g_ is the mass attenuation coefficient of gangue, and *c*_*g*_ is the mass percentage content of gangue in the mixture. For a specific mining face, *μ*_c_, *μ*_g_ and the radiation matter concentration q of gangue can be treated as constants. Assuming that the volume percentage content of coal in the mixture is v_c_, whereas that of gangue is v_g_, then

2 $${{\rm{v}}}_{{\rm{c}}}+{{\rm{v}}}_{{\rm{g}}}=1$$

3 $${{\rm{c}}}_{{\rm{c}}}+{{\rm{c}}}_{{\rm{g}}}=1$$

Let the mixture’s total volume and total mass be V and M, respectively, then

4 $$V=\frac{M{c}_{c}}{{\rho }_{c}}+\frac{M{c}_{g}}{{\rho }_{g}}.$$

Combining Equations (2~4), we have

5 $${v}_{g}=\frac{{\rho }_{c}{{\rm{c}}}_{g}}{{\rho }_{g}+({\rho }_{c}-{\rho }_{g}){c}_{g}}.$$

For a infinitely small volume dv in the mixture shown in Fig. 6, its radiation on point P is

6 $$dJ=\frac{{\rho }_{g}q{v}_{g}dv}{{r}^{2}}{e}^{-\mu r}.$$

where *dv* = *r* ^2^ *θ*sin*ϕdϕdθdr*. Perform volume integral for the entire radiation body, letting θ = 2π, ϕ = π, and r is infinitely great, then

7 $$J={\rho }_{g}q{v}_{g}{\int }_{0}^{2\pi }d\theta {\int }_{0}^{\pi }\sin \,\varphi d\varphi {\int }_{0}^{\infty }{e}^{-\mu r}dr=\frac{4\pi }{\mu }{\rho }_{g}q{v}_{g}.$$

Combining Equations (1), (5) and (7), we have

8 $$J=\frac{4\pi q{\rho }_{c}{\rho }_{g}{c}_{g}}{[{\mu }_{c}+({\mu }_{g}-{\mu }_{c})]\,[{\rho }_{g}+({\rho }_{c}-{\rho }_{g}){c}_{g}]}.$$

It can be seen from Equation (8) that the detection of radiation intensity of coal and gangue mixture is related to the density of coal and gangue, mass attenuation coefficient, content radioactive concentration of gangue. For the determined geological conditions, the density and mass attenuation coefficient of coal and gangue, and the radioactive concentration of gangue are determined in Equation (8), therefore the radiation intensity J has a one-to-one correspondence with refuse content in coal and gangue mixture, it means that we can get the refuse rate by measuring the radiation intensity of the mixture.

### Experimental analysis

In order to measure the coal and gangue radiation characteristics in fully mechanized caving mining, to verify its strong and weak law, to determine threshold and real time response of the detector and to discuss the feasibility of coal and gangue recognition technology in fully mechanized caving mining by means of natural ray, we carried out the test experiment in the laboratory.

### Experimental scheme

The experimental test method is a combination of static experiment and dynamic experiment, the coal and gangue materials used for the experiment were selected from a fully mechanized caving face of a coal mine which belongs to Shanxi Datong Coal Mine Group.

- Environment background radiation calibration: first of all, the natural background value of the experimental site was measured, and the background radiation value was obtained by the average value of a large number of samples.
- Crushed coal and gangue radiation test: gangue and coal samples were selected from a fully mechanized caving face of a coal mine which belongs to Shanxi Datong Coal Mine Group. To break the coal samples to pieces, and then successively accumulating them in an equal amount and put them under the probe for radiation measurement. And then do the same procedure for the gangue samples.
- Top coal caving simulation test: put 300 mm crushed coal in the funnel, and then tiled 500 mm crushed gangues, the detector height was reduced to 200 mm from the platform bottom plate, the platform was lifted to a tilt angle of 35°, then opened the software to detect the background radiation for a certain time, then opened the coal to let the coal and gangue run out freely, to detect the change of mixed state and the radiation intensity of coal and gangue in the stream process. When all the released materials were gangue, the change of gangue stream can be controlled through opening and closing the window, in order to observe whether the response speed of the instrument can meet the requirements of real-time response.

### Top coal caving simulation test

**Figure 8:**
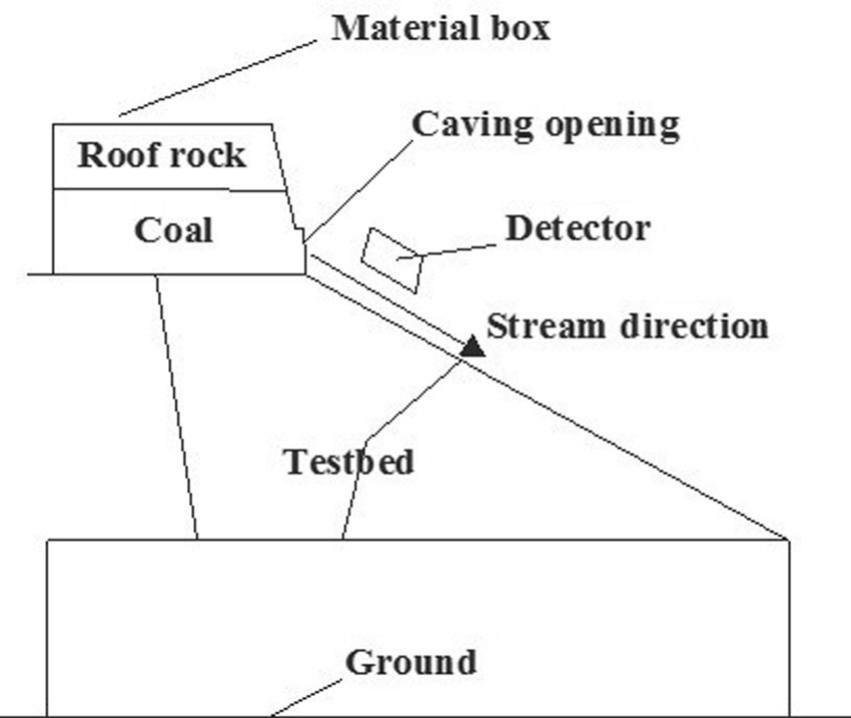
Layout schematic diagram of the model.

**Figure 9:**
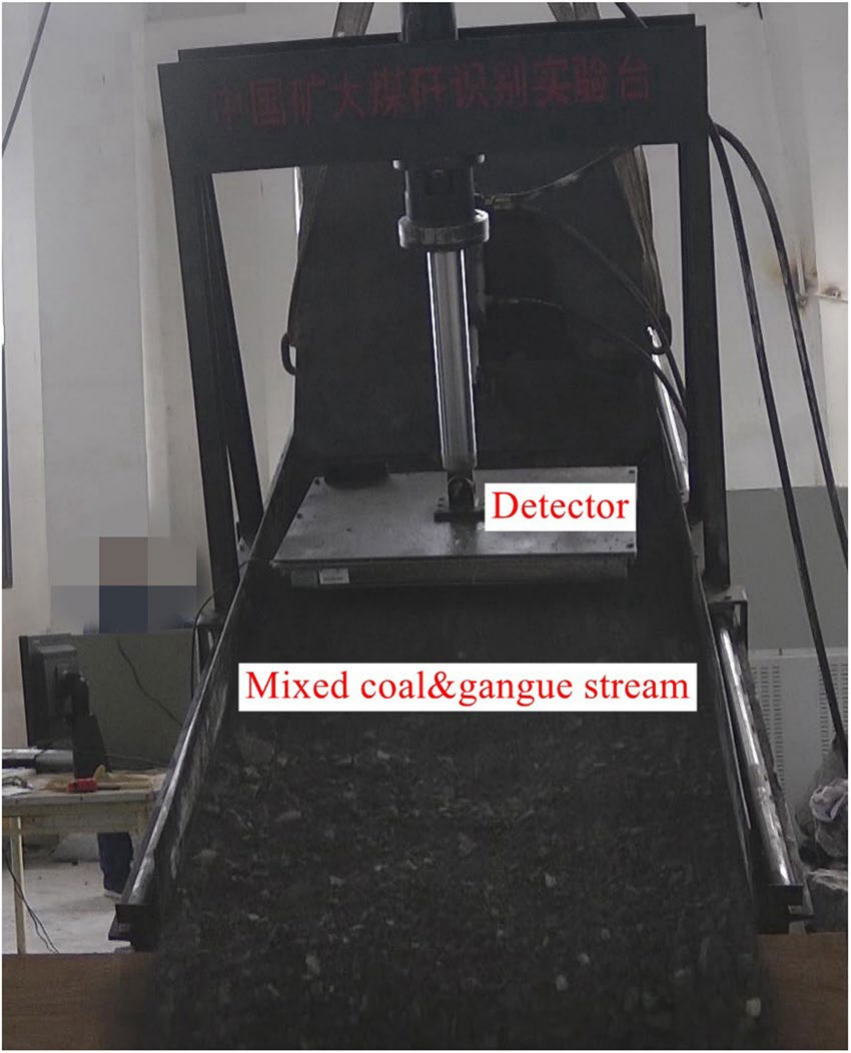
Top coal caving simulation test.

To simulate the top coal caving process by coal and gangue recognition test rig, and the radiation characteristics of coal and gangue were measured. The experiment system was show in Figs 8 and 9. 300 kg coal was first installed at the bottom of the coal discharging funnel, the funnel was tilted for 30° to let the coal has natural balance. Then the gangue was loaded until the funnel was filled. Put the funnel on the test bench and fixed it, and adjusted the distance between the probe and the platform to 10 cm. Lift the platform to the place where has 30° angle to the ground. Using the monitoring software to measure the background for one minute, then opened the funnel damper to cave the coal and monitor the data. When the data rose to the highest point, the damper was slowly closed and then opened again, to examine the response of the probe to the gangue in coal stream.
